# Tobacco use and age are associated with different morphologic features of anterior communicating artery aneurysms

**DOI:** 10.1038/s41598-021-84315-5

**Published:** 2021-02-26

**Authors:** Jian Zhang, Pui Man Rosalind Lai, Anil Can, Srinivasan Mukundan, Victor M. Castro, Dmitriy Dligach, Sean Finan, Vivian S. Gainer, Nancy A. Shadick, Guergana Savova, Shawn N. Murphy, Tianxi Cai, Scott T. Weiss, Rose Du

**Affiliations:** 1 Department of Neurosurgery, Brigham and Women’s Hospital, Harvard Medical School, 75 Francis Street, Boston, MA 02115 USA; 2 Department of Neurosurgery & Brain and Nerve Research Laboratory, The First Affiliated Hospital of Soochow University, Suzhou, Jiangsu Province China; 3 Department of Neurosurgery, Amsterdam University Medical Centers, Amsterdam, the Netherlands; 4 Department of Radiology, Brigham and Women’s Hospital, Boston, MA USA; 5 Research Information Systems and Computing, Massachusetts General Brigham, Boston, MA USA; 6 Boston Children’s Hospital Informatics Program, Boston, MA USA; 7 Department of Computer Science, Loyola University, Chicago, IL USA; 8 Division of Rheumatology, Immunology and Allergy, Brigham and Women’s Hospital, Boston, MA USA; 9 Department of Neurology, Massachusetts General Hospital, Boston, MA USA; 10 Harvard T.H. Chan School of Public Health, Boston, MA USA; 11 Channing Division of Network Medicine, Brigham and Women’s Hospital, Boston, MA USA

**Keywords:** Neurovascular disorders, Risk factors

## Abstract

We present a cohort of patients with anterior communicating artery (ACoA) aneurysms to investigate morphological characteristics and clinical factors associated with rupture of the aneurysms. 505 patients with ACoA aneurysms were identified at the Brigham and Women’s Hospital and Massachusetts General Hospital between 1990 and 2016, with available CT angiography (CTA). Three-dimensional (3D) reconstructions were performed to evaluate aneurysmal morphologic features, including location, projection, irregularity, the presence of daughter dome, height, height/width ratio, and relationships between surrounding vessels. Patient risk factors assessed included patient age, sex, tobacco use, alcohol use, and family history of aneurysms and aneurysmal subarachnoid hemorrhage. Logistic regression was used to build a predictive ACoA score for rupture. Morphologic features associated with ruptured ACoA aneurysms were the presence of a daughter dome (OR 21.4, 95% CI 10.6–43.1), smaller neck diameter (OR 0.55, 95% CI 0.42–0.71), larger aspect ratio (OR 3.57, 95% CI 2.05–6.24), larger flow angle (OR 1.03, 95% CI 1.02–1.05), and smaller ipsilateral A2-ACoA angle (OR 0.98, 95% CI 0.97–1.00). Tobacco use was predominantly associated with morphological factors intrinsic to the aneurysm that were associated with rupture while younger age was also associated with morphologic features extrinsic to the aneurysm that were associated with rupture. The ACoA score had good predictive capacity for rupture with AUC = 0.92 using the 0.632 bootstrap cross-validation for correction of overfitting bias. Ruptured ACoA aneurysms were associated with morphological features that are simple to assess using a simple scoring system. Tobacco use and younger age were predominantly associated with intrinsic and extrinsic morphological features characteristic of rupture, respectively.

## Introduction

Rupture of an intracranial aneurysm is unpredictable and can lead to significant morbidity and mortality. There is ongoing effort into understanding the natural history of aneurysm growth and factors associated with rupture to improve our clinical assessment for treatment. Anterior communicating artery (ACoA) aneurysms are amongst the most common intracranial aneurysms to present after rupture and are estimated to account for up to 30% of all intracranial aneurysms^1,2^. The advancement of computed tomography angiography (CTA) and digital subtraction angiography (DSA) has provided a greater venue to assess morphologic features that may be associated with rupture of an aneurysm. Features previously proposed to be associated with ACoA aneurysm rupture included intrinsic aneurysm characteristics, such as aneurysm size, height and projection, and transitional characteristics, such as the relationship of the aneurysm with the parent vessels^3,4^. It has been postulated that the underlying mechanisms and determinants of aneurysm ruptures are multifactorial, but are associated with hemodynamic loads, wall biomechanics, and the local aneurysm environment^5^.

In addition to intrinsic aneurysm characteristics, patients’ clinical factors, such as tobacco use and hypertension, are well-established risk factors associated with intracranial aneurysm rupture^6,7^. Differences in aneurysm morphology and size have been proposed between smoking and non-smoking patients^8,9^. It is thought that nicotine promotes local inflammation and sustained angiogenesis, resulting in downstream changes and alteration of aneurysm morphology^10^. Here, we present a large cohort of 505 patients with anterior communicating artery (ACoA) aneurysms to assess morphological features associated with ruptured aneurysms, as well as, the association of patient characteristics, including age and tobacco use, with those morphological features.

## Methods

### Patient selection

Patients diagnosed with intracranial aneurysms at the Brigham and Women’s Hospital (BWH) and Massachusetts General Hospital (MGH) between 1990–2016 were identified using natural language processing (NLP), as well as prospectively, as previously described^11^. Manual medical record review was performed to confirm the diagnosis of a cerebral artery aneurysm. Briefly, both codified and NLP data were used to identify an initial set of patients with potential aneurysms from the Research Patient Data Registry (RPDR), the research version of our electronic medical record, 5,589 patients were initially identified^11^. 727 of these patients were also seen on clinical presentation from 2007–2013 with prospectively collected data. Additionally, 474 patients with prospectively collected data who were seen on clinical presentation from 2013–2016, were also included^11^. 4701 patients with definite saccular aneurysms were identified by manually reviewing (AC and RD) the electronic medical records of all 6063 patients^11^. Patients with non-saccular aneurysms, aneurysms associated with arteriovenous malformations, or aneurysms that lacked preoperative CT angiography (CTA) were excluded from this study.

505 patients with anterior communicating (ACoA) aneurysms were included in this study. CT angiography (CTA) images were obtained using mi2b2 open-source software to comply with research privacy requirements^12^. Relevant demographic and clinical data, including age, sex, hypertension, current tobacco use, heavy tobacco use > 1 pack per day, years since quitting tobacco, alcohol use, hypertension, atrial fibrillation, history of ischemic stroke, coronary artery disease, history of myocardial infarction, family history of intracranial aneurysms and aneurysmal subarachnoid hemorrhage, and aspirin anticoagulant, and antihyperlipidemic agent use at diagnosis, were retrieved from the medical records. This study was approved by the Partners Human Research Committee which also waived the requirement for informed consent. All procedures performed were in accordance with the ethical standards of the institutional review board and with the 1964 Helsinki declaration and its later amendments or comparable ethical standards.

### Reconstruction of 3-dimensional models

**Figure 1:**
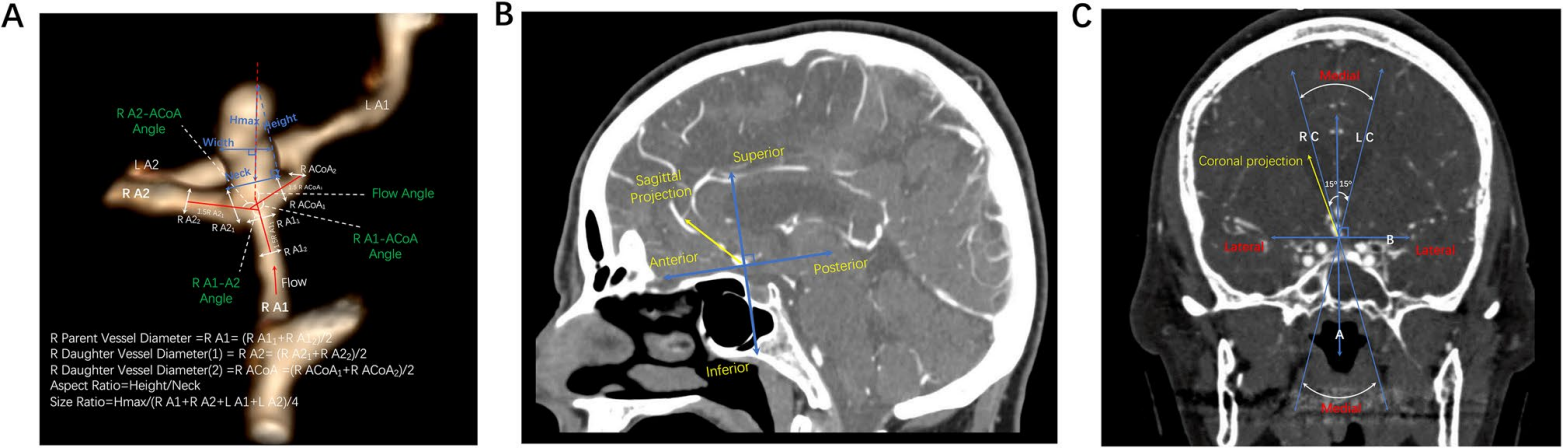
(**A**) Illustration of morphological parameters for anterior communicating artery (ACoA) aneurysm. (**B**) Projection of ACoA aneurysm based on sagittal view. Horizontal line is parallel to the anterior skull base. (**C**) Location of ACoA aneurysm based on coronal projections. Medial location is defined as projections within 15° of midline, between lines RC and LC. Image was partially created by Vitrea Advanced Visualization software (version 6.9.68.1).

Three-dimensional (3D) models of aneurysms and their surrounding vasculature were generated from DICOM (Digital Images and Communication in Medicine) format using preoperative CTA by the Vitrea Advanced Visualization software (version 6.9.68.1, Vital Images, Minnetonka, MN)^13^. Images were evaluated by an attending neurosurgeon (JZ) with verification by a second neurosurgeon (RD) when needed. Detailed protocol on the construction of the 3-dimensional images and aneurysm measurements were previously described^14^. Intrinsic, transitional and extrinsic morphological parameters of the aneurysms were studied to assess their risk for rupture (Fig. 1). Intrinsic characteristics are those that involve the aneurysm only, extrinsic characteristics involve only the surrounding vessels, and transitional ones involve both the aneurysm and the surrounding vessels^15^.

Aneurysms were assessed for their location (middle, left or right), projection (anterior, posterior, superior or inferior), the smoothness of the wall (smooth or irregular), and the presence of a daughter dome. The maximum height of the aneurysm was measured from the center of the aneurysm neck to the greatest distance on the aneurysm dome. Maximum perpendicular height was measured as the distance from the center of the aneurysm neck to the perpendicular height of the aneurysm dome. Neck diameter was defined as the width of the aneurysm neck. Aspect ratio was calculated as the ratio of maximum perpendicular height to the neck diameter. In addition, the maximum width of the aneurysm was measured, and a height/width ratio was calculated by dividing the maximum perpendicular height by the width.

Extrinsic parameters of the aneurysms involving the surrounding vasculature were also assessed. The average A1 length and the diameters of the ACoA, ipsilateral A1, ipsilateral A2, contralateral A1, and contralateral A2 were measured. Mean diameter of the vessels was measured by averaging the diameter of the cross-section of a vessel just proximal to the neck of the aneurysm, and the diameter of the cross-section at 1.5 times from the neck of the aneurysm. Anatomical variations were also noted, including ipsilateral hypoplastic A1, and ipsilateral or contralateral aplastic A1. The angles between ipsilateral A1-A2, ipsilateral A1-ACoA, ipsilateral A2-ACoA, contralateral A1–A2, contralateral A1-ACoA, and contralateral A2-ACoA were also measured.

Transitional parameters evaluated included size ratio and flow angle. Size ratio was defined as the ratio between the maximum aneurysm height and the mean vessel diameter of all branches associated with the aneurysm. Flow angle is the angle between the maximum height of the aneurysm and the parent vessel.

### Statistical analysis

Demographic baseline characteristics between ruptured and unruptured ACoA aneurysms were evaluated using the student t-test for continuous variables and the Pearson’s chi-square test for categorical variables. Univariate and multivariate logistic regression models were used to study different morphological parameters on the rupture status of ACoA aneurysms, with a backward elimination procedure to identify significant confounders with a cut-off value of p = 0.1 for the initial selection of variables. Additional univariate and multivariate analyses were performed to study patient characteristics and risk factors and their associations with morphologic parameters that met statistical significance.

A predictive score for rupture was created using the results of the multivariate model including covariates with p < 0.05. Points were allocated according to OR, with -3 points for OR < 1/2, -2 points for OR [1/2,2/3), − 1 point for OR [2/3,1.0), 1 point for OR [1.0,1.5), 2 points for OR [1.5,2.0), 3 points for OR [2.0,3.0), and 4 points for OR ≥ 2.0. The thresholds used for continuous variables were the mean value between the ruptured and unruptured groups. The predictive capacity of the score was evaluated by using a logistic regression with lasso penalty. The area under the receiver operating curve (AUC) was obtained using the 0.632 bootstrap cross-validation for correction of overfitting bias via the *ROC632*^16^ package in R^17^ (version 3.6.2). The goodness-of-fit of the model was evaluated with the Hosmer–Lemeshow test using quartiles for risk intervals. Statistical analyses were performed using the Stata statistical software (version 14, StatCorp. College Station, TX) and R^17^ (version 3.6.2). A sensitivity analysis was performed for the predictive score by removing variables that may be affected by the rupture process such as the presence of a daughter dome. A p value of less than 0.5 was considered statistically significant.

## Results

### Baseline characteristics of ruptured and unruptured ACoA aneurysms

505 patients with ACoA aneurysms were identified in this study. Table 1 shows the demographics of the study population. The mean age at presentation was 57 ± 13 years, with females representing 62% of the cohort. Patients with ruptured aneurysms presented earlier than those with unruptured aneurysms (54 ± 13 years vs 61 ± 12 years, p < 0.01), with a higher rate of tobacco use (38% vs 28%, p < 0.01) and alcohol use (57% vs 45%, p = 0.01). Female sex, hypertension, family history of SAH and family history of intracranial aneurysms were not different between the ruptured and unruptured cohorts.

**Table 1:** Baseline demographics and aneurysm characteristics of patients with unruptured and ruptured anterior communicating (ACoA) aneurysms (N = 505).

	Unruptured ACoA aneurysms (N = 274)	Ruptured ACoA aneurysms (N = 231)	Total (N = 505)	P value
**Patient characteristics**				
Age at presentation (mean ± SD)	61 ± 12	54 ± 13	57 ± 13	** < 0.01**
Female (%)	174 (64)	135 (58)	309 (62)	0.25
Current tobacco use (%)	74 (28)	95 (38)	169 (34)	** < 0.01**
Tobacco use > 1 pack per day (%)	16 (5.8)	21 (9.1)	37 (7.3)	0.16
Years since quit tobacco (SD)	9.5 (22.4)	21.5 (26.9)	20.6 (26.7)	0.10
Current alcohol use (%)	114 (45)	122 (57)	236 (51)	**0.01**
Hypertension (%)	153 (56)	124 (54)	277 (55)	0.63
Atrial fibrillation*	0 (0)	5 (2.4)	5 (2.2)	0.51
History of ischemic stroke*	0 (0)	16 (7.6)	16 (7.0)	0.23
Coronary artery disease*	0 (0)	11 (5.2)	11 (4.8)	0.32
History of myocardial infarction (%)*	0 (0)	8 (3.8)	8 (3.5)	0.40
Family history of SAH (%)	24 (9)	15 (7)	39 (8)	0.32
Family history of intracranial aneurysm (%)	47 (17)	28 (12)	75 (15)	0.10
Aspirin use at diagnosis (%)*	0 (0)	26 (12.3)	26 (11.4)	0.11
Anticoagulant use at diagnosis (%)*	0 (0)	5 (2.4)	5 (2.2)	0.51
Antihyperlipidemic agent use at diagnosis (%)*	1 (5.6)	36 (17.1)	37 (16.2)	0.20
**Intrinsic morphological parameters**				
Projection (%)				**0.04**
Anterior (Ref)	222 (81)	202 (87)	424 (84)	
Posterior	15 (5.5)	13 (5.6)	28 (5.5)	
Superior	19 (6.9)	9 (3.9)	28 (5.5)	
Inferior	18 (6.6)	7 (3.0)	25 (5.0)	
**Location (%)**				0.23
Medial (Ref)	26 (9.5)	17 (7.4)	43 (8.5)	
Left	149 (54)	120 (52)	269 (53)	
Right	99 (36)	94 (41)	193 (38)	
Irregularity (%)	46 (18)	125 (54)	171 (34)	** < 0.01**
Daughter dome (%)	40 (15)	160 (69)	200 (40)	** < 0.01**
Maximum height (mm ± SD)	4.7 ± 2.6	5.7 ± 2.6	5.1 ± 2.6	** < 0.01**
Perpendicular height (mm ± SD)	4.3 ± 2.4	5.2 ± 2.5	4.7 ± 2.5	** < 0.01**
Neck diameter (mm ± SD)	3.9 ± 1.4	3.3 ± 1.3	3.6 ± 1.4	** < 0.01**
Width (mm ± SD)	4.5 ± 2.2	4.5 ± 2.3	4.5 ± 2.3	0.98
Height/width ratio	0.95 ± 0.3	1.2 ± 0.4	1.1 ± 0.3	** < 0.01**
Aspect ratio	1.1 ± 0.5	1.6 ± 0.6	1.3 ± 0.6	** < 0.01**
**Transitional morphological parameters**				
Size ratio	0.73 ± 0.4	0.92 ± 0.4	0.82 ± 0.4	** < 0.01**
Flow angle	125 ± 21	136 ± 22	130 ± 22	** < 0.01**
**Extrinsic morphological parameters**				
Pre-aneurysm A1 length (mm)	16 ± 2.3	16 ± 2.2	16 ± 2.2	0.67
ACoA diameter (mm)	1.6 ± 0.5	1.6 ± 0.5	1.6 ± 0.5	0.50
Ipsilateral A1 diameter (mm)	1.9 ± 0.4	1.8 ± 0.4	1.9 ± 0.4	** < 0.01**
Ipsilateral A2 diameter (mm)	1.9 ± 0.4	1.8 ± 0.4	1.8 ± 0.4	** < 0.01**
Contralateral A1 diameter (mm)	0.98 ± 0.7	0.95 ± 0.6	0.97 ± 0.6	0.34
Contralateral A2 diameter (mm)	1.9 ± 0.4	1.8 ± 0.4	1.8 ± 0.4	0.11
Hypoplastic A1	111 (41)	85 (37)	196 (39)	0.39
Aplastic A1	62 (23)	50 (22)	112 (22)	0.79
Ipsilateral A1-A2 angle	99 ± 25	105 ± 1.4	102 ± 1.0	** < 0.01**
Ipsilateral A1-ACoA angle	103 ± 22	100 ± 20	102 ± 21	0.06
Ipsilateral A2-ACoA angle	131 ± 27	125 ± 21	128 ± 24	**0.01**
Contralateral A1-A2 angle	111 ± 24	116 ± 22	113 ± 24	0.09
Contralateral A1-ACoA angle	109 ± 21	110 ± 21	109 ± 21	0.93
Contralateral A2-ACoA angle	116 ± 22	111 ± 20	113 ± 21	**0.04**

*ACoA* anterior communicating artery, *Ref* reference, *SAH* subarachnoid hemorrhage, *SD* standard deviation.

*Available data in 229 patients. Percentages calculated based on available data.

Further analyses for association between patient characteristics demonstrated an inverse relationship between tobacco use and age (OR0.96 [95% CI 0.94–0.97], p < 0.01), and an association with tobacco use and alcohol use (OR1.33 [95% CI 1.1–1.6], p < 0.01). The associations between tobacco use and aspirin use (OR1.32 [95% CI 0.58–3.0], p = 0.51), anticoagulant use (OR1.94 [95% CI 0.32–11.9] p = 0.47), and antihyperlipidemic agent use (OR0.97 [95% CI 0.47–1.97], p = 0.93) were not significant.

Table 1 illustrates baseline aneurysm characteristics of patients with unruptured and ruptured ACoA aneurysms. For intrinsic morphological parameters, ruptured aneurysms tend to be anteriorly projecting, had a larger maximal height, perpendicular height, height/width ratio, aspect ratio, but a smaller neck diameter. Both size ratio and flow angle were larger in ruptured aneurysms. For extrinsic parameters, ruptured aneurysms were associated with larger ipsilateral A1-A2 angles, smaller ipsilateral A1 and A2 diameters, and smaller ipsilateral and contralateral A2-ACoA angles.

### Morphological features associated with ruptured ACoA aneurysms

Table 2 illustrates intrinsic, transitional, and extrinsic aneurysm characteristics associated with rupture. Univariate analyses showed an association of rupture with the presence of irregular domes and daughter domes, larger maximal height, perpendicular height, height/width ratio, aspect ratio, ipsilateral A1–A2 angle, flow angle, and size ratio. Aneurysm rupture was inversely associated with neck diameter, ipsilateral A1 diameter, ipsilateral A2 diameter, and ipsilateral A2-ACoA angle.

**Table 2:** Univariate and multivariate regression models for rupture of anterior communicating (ACoA) aneurysms.

Variables	Univariable		Multivariable	
	OR (95% CI)	P value	OR (95% CI)	P value
**Intrinsic morphological parameters**				
**Projection**				
Anterior (Ref)	–	–		
Posterior	0.95 (0.44–2.05)	0.90		
Superior	0.52 (0.23–1.18)	0.12		
Inferior	0.43 (0.17–1.04)	0.06		
Location				
Medial (Ref)	–	–		
Left	1.23 (0.64–2.4)	0.53		
Right	1.45 (0.74–2.8)	0.28		
Irregularity	**5.84 (3.88–8.80)**	** < 0.01**		
Daughter dome	**13.2 (8.52–20.4)**	** < 0.01**	**21.4 (10.6–43.1)**	** < 0.01**
Maximum height (mm)	**1.17 (1.09–1.25)**	** < 0.01**		
Perpendicular height (mm)	**1.17 (1.08–1.26)**	** < 0.01**		
Neck diameter (mm)	**0.74 (0.64–0.85)**	** < 0.01**	**0.55 (0.42–0.71)**	** < 0.01**
Width (mm)	1.00 (0.93–1.08)	0.94		
Height/width ratio	**13.2 (6.69–26.2)**	** < 0.01**		
Aspect ratio	**6.75 (4.39–10.4)**	** < 0.01**	**3.57 (2.05–6.24)**	** < 0.01**
**Transitional morphological parameters**				
Size ratio	**2.73 (1.78–4.17)**	** < 0.01**		
Flow angle	**1.02 (1.02–1.03)**	** < 0.01**	**1.03 (1.02–1.05)**	** < 0.01**
**Extrinsic morphological parameters**				
Pre-aneurysm A1 length (mm)	0.97 (0.90–1.05)	0.47		
ACoA diameter (mm)	0.84 (0.54–1.29)	0.42		
Ipsilateral A1 diameter (mm)	**0.43 (0.27–0.69)**	** < 0.01**		
Ipsilateral A2 diameter (mm)	**0.56 (0.36–0.86)**	** < 0.01**		
Contralateral A1 diameter (mm)	0.91 (0.69–1.20)	0.50		
Contralateral A2 diameter (mm)	0.70 (0.43–1.12)	0.13		
Hypoplastic A1	0.85 (0.60–1.23)	0.39		
Aplastic A1	0.94 (0.62–1.44)	0.79		
Ipsilateral A1-A2 angle	**1.01 (1.00–1.02)**	** < 0.01**		
Ipsilateral A1-ACoA angle	0.99 (0.99–1.00)	0.22		
Ipsilateral A2-ACoA angle	**0.99 (0.98–1.00)**	**0.02**	**0.98 (0.97–1.00)**	**0.01**
Contralateral A1-A2 angle	1.01 (1.00–1.02)	0.04	0.99 (0.98–1.00)	0.06
Contralateral A1-ACoA angle	1.00 (0.99–1.01)	0.95		
Contralateral A2-ACoA angle	0.99 (0.98–1.00)	0.03		

*Ref* reference.

After multivariate adjustment, the presence of a daughter dome (OR21.4 [95% CI 10.6–43.1]), larger aspect ratio (OR3.57 [95% CI 2.05–6.24]), smaller neck diameter (OR0.55 [95% CI 0.42–0.71]), larger flow angle (OR1.03 [95% CI 1.02–1.05]), and smaller ipsilateral A2-ACoA angle (OR0.98 [95% CI 0.97–1.00]) remained associated with rupture status.

### Patient risk factors associated with morphological features

Morphological features associated with ruptured ACoA on univariate analyses were subsequently assessed to determine their association with clinical risk factors (Table 3 and Supplemental Tables 1–3). In the univariate analyses, age at diagnosis was associated with larger neck diameter, ipsilateral A1 diameter, ipsilateral A2 diameter, ipsilateral A2-ACoA angle, and inversely associated with aspect ratio, height/width ratio, ipsilateral A1–A2 angle, contralateral A1–A2 angle, flow angle, and the presence of daughter domes and irregularity. Active tobacco use was associated with larger maximal height, perpendicular height, aspect ratio, height/width ratio and size ratio on univariate analyses.

**Table 3:** Multivariate analyses of morphological characteristics.

	Age at diagnosis	Current tobacco use	Years since quit tobacco	Current alcohol use	Female	Hypertension	History of ischemic stroke	Coronary artery disease	Family history of SAH	Family history of aneurysms
**Intrinsic morphological parameters**										
Maximum height		1.1			− 0.79					
Perpendicular height		1.1			− 0.74					
**Aspect ratio**		0.22								
Height/width							0.02			
**Neck diameter**	0.02								− 0.42	
Irregularity	0.98 OR									
**Daughter dome**	0.98 OR									
**Transitional morphological parameters**										
Size ratio		0.19			− 0.18					
**Flow angle**	− 0.24									
**Extrinsic morphological parameters**										
Ipsilateral A1			0.002					0.27		
Ipsilateral A2				− 0.10				0.29		
Ipsilateral A1-A2 angle	− 0.21				5.8					
**Ipsilateral A2-ACoA angle**			0.11			−				
Contralateral A1-A2 angle	− 0.34									

Regression coefficients or odds ratios (OR) of significant factors are shown. Morphological characteristics significantly associated with rupture in multivariate analysis are bolded. See Supplemental Tables for complete results.

*ACoA* anterior communicating artery.

After multivariate adjustment, age at diagnosis remains associated with larger neck diameter (β0.02 [95% CI 0.02–0.03]), and inversely associated with ipsilateral A1-A2 angle (β− 0.21 [95% CI − 0.41 to − 0.01]), contralateral A1–A2 angle (β− 0.34 [95% CI − 0.57 to − 0.12]), flow angle (β− 0.24 [95% CI − 0.39 to 0.078]). and the presence of irregular domes (OR0.98 [95% CI 0.96–0.99]) and daughter domes (OR 0.98 [0.96–0.99]). Current tobacco use was associated with larger maximal height (β1.1 [95% CI 0.44–1.8]), perpendicular height (β1.1 [95% CI 0.38–1.8]), aspect ratio (β0.22 [95% CI 0.06–0.37]), and size ratio (β0.19 [95% CI 0.06–0.31]). Other associations with female sex, years since quitting smoking, alcohol use, hypertension, coronary artery disease, history of ischemic stroke, and family history of aneurysms and SAH were also found.

### Predictive ACoA score

**Figure 2:**
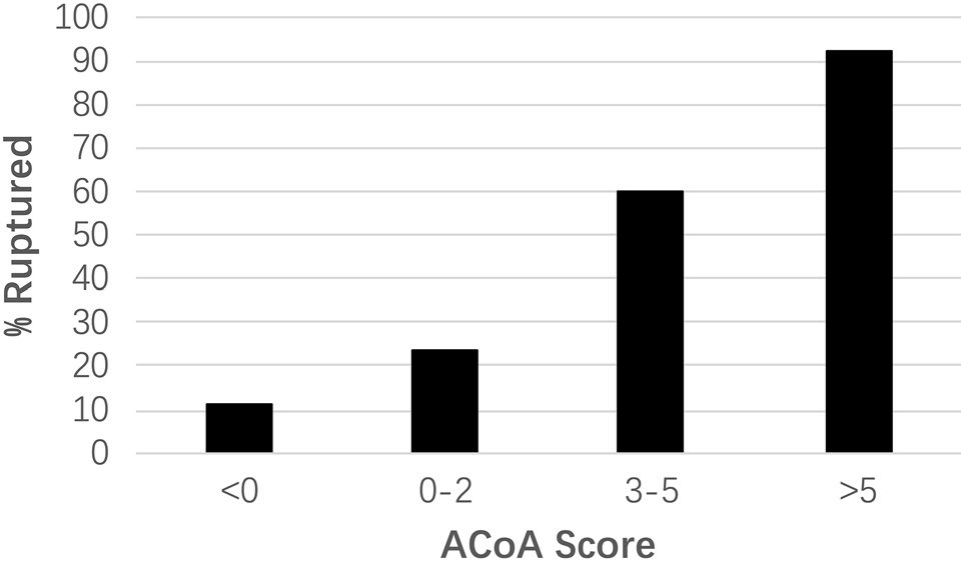
Percent ruptured aneurysms stratified by predictive anterior communicating artery aneurysm score defined in Table 4.

The predictive ACoA score obtained from the multivariable regression model is shown in Table 4. The AUC of the ACoA score from the 0.632 bootstrap cross-validation was 0.92. The p-value for the Hosmer–Lemeshow test was 0.15, signifying good fit. The proportion of ruptured aneurysms stratified by ACoA score is shown in Fig. 2.

**Table 4:** Anterior communicating artery (ACoA) score for prediction of rupture.

Points	Characteristics
− 2	Neck diameter > 3.6 mm
− 1	Ipsilateral A2-ACoA angle > 128
1	Flow angle > 130
4	Presence of daughter dome
4	Aspect ratio > 1.3
− 3 to 9	Range of possible points

A sensitivity analysis was performed by removing the presence of a daughter dome as that may occur as a result of the rupture process. The predictive score using this reduced model is shown in Supplemental Table 4. The AUC from the 0.632 bootstrap cross-validation was 0.89. The p-value for the Hosmer–Lemeshow test was 0.55. The proportion of ruptured aneurysms stratified by the reduced score is shown in Supplemental Fig. 1.

## Discussion

In this study, we present a cohort of 505 patients with ACoA aneurysms, and studied aneurysm morphological factors and patient characteristics associated with aneurysm rupture. We demonstrated that the presence of a daughter dome, smaller neck diameter, larger aspect ratio, larger flow angle, and smaller ipsilateral A2-ACoA angle were associated with ACoA aneurysm rupture. We also demonstrated that tobacco use was predominantly associated with intrinsic aneurysm characteristics associated with rupture including larger aspect ratio. In contrast, younger age was also associated with extrinsic aneurysm characteristics associated with rupture, including smaller neck diameter, the presence of irregularity and a daughter dome, larger flow angle, and larger ipsilateral A1–A2 angle and contralateral A1–A2 angle.

### Morphological risk factors

We identified several intrinsic morphological features that were associated with rupture of ACoA aneurysms. We found the presence of a daughter dome to have the highest association with rupture risk. This is in support of prior studies which demonstrated daughter sacs to be highly associated with rupture status^18–20^. We also found aneurysm rupture to be associated with a larger aspect ratio and a smaller neck diameter. While aspect ratio and similar parameters such as height/width ratio have been found to be significant in prior studies^21^, neck diameter has not previously been found to be significant and may be due to our large sample size. A smaller neck diameter may affect the location and size of the impingement zone, size and location of the inflow jet, and the area of low wall shear stress^22^. These changes may contribute to the increased rupture risk. We did not find an association of aneurysm rupture with projection or the location of the ACoA aneurysm.

Our analysis of the extrinsic and transitional morphological features demonstrated larger flow angle and smaller ipsilateral A2-ACoA angle to be associated with rupture. Flow angle reflects the relationship between the aneurysm and the parent vessel and has previously been described to be associated with rupture^23^, but not all studies found this association^24,25^. Computational fluid dynamics modeling has demonstrated that larger flow angles resulted in higher flow velocity and wall shear stress, which may explain the association of flow angle with aneurysm rupture^27^. Our study also found a smaller ipsilateral A2-ACoA angle to be associated with rupture which has not previously been shown. The A2-ACoA angle has been shown to be inversely associated with ipsilateral A1 and A2 diameter, which was thought to explain its contribution to hemodynamic stress, but we did not find this association^26^.

### Patient risk factors

Analysis of patient characteristics demonstrated tobacco use to be predominantly associated with intrinsic morphological features of ruptured aneurysms. Specifically, tobacco use was associated with larger maximal and perpendicular heights, aspect ratio, and size ratio. Smoking is a known independent risk factor for aneurysm formation and rupture, with a dose–response curve with the duration of smoking and increased rupture risk^6,10,27–29^. Our study further demonstrated that ACoA aneurysms in patients with active tobacco use have morphological features associated with rupture. It can be postulated that tobacco use may alter local aneurysm environment and architecture through its inflammatory and downstream pathways, resulting in morphological changes associated with higher rupture risk^10^.

In our series, patients with ruptured aneurysms presented at a younger age and moreover, younger age was associated with intrinsic, transitional, and extrinsic morphological features associated with aneurysm rupture, namely, the presence of irregular domes and daughter domes, smaller neck diameter, larger flow angle, and larger ipsilateral and contralateral A1–A2 angles. In contrast with tobacco use, these morphological features also include those that are extrinsic to the aneurysm. While older age is considered a risk factor for aneurysm rupture^30^, we found an opposite effect where ruptured ACoA aneurysms occurred at a younger age than unruptured ACoA aneurysms, consistent with prior observations^31–33^. This increase in rupture among younger patients may be explained by the changes to the surrounding vasculature, such as the A1–A2 angles, that occur with age.

### Limitations

While an association with morphological patterns and rupture status can be investigated, these factors do not necessarily predict rupture risk as these patterns may reflect post-rupture changes. However, the extrinsic morphological factors and other intrinsic parameters such as neck diameters are unlikely to alter with aneurysm rupture and can serve as more robust parameters for risk prediction. To address the potential post-rupture changes, we have performed a sensitivity analysis with the presence of a daughter dome removed from our predictive score and achieved comparable results. A final limitation is that our prediction model is not independently validated. Despite these limitations, this is the largest series of ACoA aneurysms to our knowledge that assesses morphological features and patient risk factors associated with rupture.

## Conclusion

We found that the presence of a daughter dome, smaller neck diameter, larger aspect ratio, larger flow angle and smaller ipsilateral A2-ACoA angle were associated with rupture. In addition, tobacco use and younger age were associated with predominantly intrinsic and extrinsic morphological parameters associated with rupture, respectively. The assessment of these morphologic features using a simple scoring system in addition to clinical risk factors may help in the risk assessment and management of patients with ACoA aneurysms.

## Supplementary Information

Supplementary Information

## Data Availability

The datasets generated during and/or analysed during the current study are available from the corresponding author on reasonable request.
