# Supplementary material for: Tobacco use and age are associated with different morphologic features of anterior communicating artery aneurysms
Source: Sci Rep. 2021 Feb 26;11:4791. doi: 10.1038/s41598-021-84315-5 (PMC7910488; doi:10.1038/s41598-021-84315-5)
Supplement: Supplementary file 1 — Supplementary Information [file 41598_2021_84315_MOESM1_ESM.docx]

**Tobacco use and age are associated with different morphologic features of anterior communicating artery aneurysms**

Jian Zhang, MD^1,2,3^ , Pui Man Rosalind Lai, MD^1,3^, Anil Can, MD^1,4^, Srinivasan Mukundan, Jr., MD, PhD^5^, Victor M. Castro, MS^6^, Dmitriy Dligach, PhD^7,8^, Sean Finan, BS^7^, Vivian S. Gainer, MS^6^, Nancy A. Shadick, MD, MPH^9^, Guergana Savova, PhD^7^, Shawn N. Murphy, MD, PhD^6,10^, Tianxi Cai, PhD^11^, Scott T. Weiss, MD, MS^12^, Rose Du MD, PhD^1,12^

^1^Department of Neurosurgery, Brigham and Women’s Hospital, Harvard Medical School, Boston, MA, USA

^2^Department of Neurosurgery & Brain and Nerve Research Laboratory, The First Affiliated Hospital of Soochow University, Jiangsu Province, China

^3^These authors contributed equally

^4^Department of Neurosurgery, Amsterdam University Medical Centers, Amsterdam, the Netherlands.

^5^Department of Radiology, Brigham and Women’s Hospital, Boston, MA

^6^Research Information Systems and Computing, Massachusetts General Brigham, Boston, MA

^7^Boston Children’s Hospital Informatics Program, Boston, MA

^8^Department of Computer Science, Loyola University, Chicago, IL

^9^Division of Rheumatology, Immunology and Allergy, Brigham and Women’s Hospital, Boston, MA

^10^Department of Neurology, Massachusetts General Hospital, Boston, MA

^11^Biostatistics, Harvard T.H. Chan School of Public Health, Boston, MA

^12^Channing Division of Network Medicine, Brigham and Women’s Hospital, Boston, MA

***Corresponding author**

Rose Du, M.D., Ph.D.

Department of Neurosurgery

Brigham and Women’s Hospital

75 Francis Street

Boston, MA 02115

Phone: 617-732-6600

Fax: 617-713-3050

Email: [rdu@bwh.harvard.edu](mailto:rdu@bwh.harvard.edu)

**Supplemental Table 1**. Univariate and multivariate regression analyses for intrinsic properties of anterior communicating artery (ACoA) aneurysms.

|  | **Univariate** | | **Multivariate** | |
| --- | --- | --- | --- | --- |
|  | **Coef [95%CI]** | **P value** | **Coef [95% CI]** | **P value** |
| **Maximal height** |  |  |  |  |
| Age at diagnosis | 0.003 [-0.02-0.02] | 0.73 |  |  |
| Current alcohol use | -0.076 [-0.56-0.40] | 0.76 |  |  |
| Current tobacco use | 1.0 [0.54-1.5] | <0.01 | 1.14 [0.44-1.84] | **<0.01** |
| Tobacco use >1 pack per day | 1.24 [0.36-2.13] | <0.01 |  |  |
| Years since quit tobacco | -0.01 [-0.03-1.0×10^-4^] | 0.052 |  |  |
| Female | -0.5 [-0.97- -0.037] | 0.03 | -0.79 [-1.50- -0.79] | **0.03** |
| Hypertension | 0.27 [-0.19-0.73] | 0.25 |  |  |
| Atrial fibrillation | 0.29 [-1.99-2.57] | 0.80 |  |  |
| History of ischemic stroke | 0.74 [-0.57-2.04] | 0.27 |  |  |
| Coronary artery disease | 0.95 [-0.60-2.51] | 0.23 |  |  |
| History of myocardial infarction | 0.68 [-1.13-2.50] | 0.46 |  |  |
| Family history of SAH | -0.85 [-1.7-0.0038] | 0.05 |  |  |
| Family history of aneurysms | -0.38 [-1.0-0.26] | 0.24 |  |  |
| Aspirin use at diagnosis | -0.22 [-1.27-0.84] | 0.69 |  |  |
| Anticoagulant use at diagnosis | -0.45 [-2.73-1.84] | 0.70 |  |  |
| Antihyperlipidemic agent use at diagnosis | -0.04 [-0.95-0.86] | 0.92 |  |  |
|  | | | | |
| **Perpendicular height** |  |  |  |  |
| Age at diagnosis | 0.003 [-0.01-0.02] | 0.70 |  |  |
| Current alcohol use | -0.09 [-0.55-0.37] | 0.70 |  |  |
| Current tobacco use | 0.97 [0.50-1.43] | <0.01 | 1.09 [0.38-1.80] | **<0.01** |
| Tobacco use >1 pack per day | 1.20 [0.36-2.05] | <0.01 |  |  |
| Years since quit tobacco | -0.01 [-0.03-0.001] | 0.07 |  |  |
| Female | -0.46 [-0.91- -0.01] | 0.04 | -0.74 [-1.46- -0.02] | **0.04** |
| Hypertension | 0.34 [-0.10-0.78] | 0.13 |  |  |
| Atrial fibrillation | 0.26 [-1.98-2.50] | 0.82 |  |  |
| History of ischemic stroke | 0.71 [-0.58-1.99] | 0.28 |  |  |
| Coronary artery disease | 1.05 [-0.48-2.57] | 0.18 |  |  |
| History of myocardial infarction | 0.84 [-0.94-2.63] | 0.35 |  |  |
| Family history of SAH | -0.64 [-1.5-0.18] | 0.13 |  |  |
| Family history of aneurysms | -0.29 [-0.90-0.33] | 0.36 |  |  |
| Aspirin use at diagnosis | -0.04 [-1.07-0.99] | 0.94 |  |  |
| Anticoagulant use at diagnosis | -0.15 [-2.39-2.10] | 0.90 |  |  |
| Antihyperlipidemic agent use at diagnosis | -0.06 [-0.95-0.83] | 0.89 |  |  |
|  | | | | |
| **Aspect Ratio** |  |  |  |  |
| Age at diagnosis | -0.008 [-0.01- -0.004] | <0.01 |  |  |
| Current alcohol use | 0.036 [-0.07-0.14] | 0.49 |  |  |
| Current tobacco use | 0.27 [0.17-0.38] | <0.01 | 0.22 [0.06-0.37] | **<0.01** |
| Tobacco use >1 pack per day | 0.18 [-0.01-0.37] | 0.06 |  |  |
| Years since quit tobacco | -0.002 [-0.005-7.7×10^-4^] | 0.14 |  |  |
| Female | -0.08 [-0.18-0.02] | 0.12 |  |  |
| Hypertension | 0.01 [-0.09-0.11] | 0.82 |  |  |
| Atrial fibrillation | 0.04 [-0.47-0.55] | 0.88 |  |  |
| History of ischemic stroke | 0.28 [-0.01-0.57] | 0.06 | 0.27 [-0.02-0.57] | 0.07 |
| Coronary artery disease | 0.25 [-0.10-0.60] | 0.15 |  |  |
| History of myocardial infarction | 0.35 [-0.06-0.75] | 0.09 |  |  |
| Family history of SAH | -0.04 [-0.2-0.15] | 0.68 |  |  |
| Family history of aneurysms | -0.03 [-0.2-0.11] | 0.71 |  |  |
| Aspirin use at diagnosis | -0.02 [-0.26-0.22] | 0.87 |  |  |
| Anticoagulant use at diagnosis | 0.21 [-0.31-0.72] | 0.43 |  |  |
| Antihyperlipidemic agent use at diagnosis | 0.08 [-0.13-0.28] | 0.47 |  |  |
|  | | | | |
| **Height/width ratio** |  |  |  |  |
| Age at diagnosis | -0.003 [-0.006- -0.001] | <0.01 |  |  |
| Current alcohol use | 0.02 [-0.04-0.08] | 0.54 |  |  |
| Current tobacco use | 0.11 [0.05-0.18] | <0.01 |  |  |
| Tobacco use >1 pack per day | -0.01 [-0.13-0.10] | 0.85 |  |  |
| Years since quit tobacco | 4.7×10^-4^ [-0.002-0.002] | 0.64 |  |  |
| Female | -0.03 [-0.09-0.03] | 0.32 |  |  |
| Hypertension | -0.009 [-0.07-0.05] | 0.76 |  |  |
| Atrial fibrillation | 0.17 [-0.15-0.50] | 0.30 |  |  |
| History of ischemic stroke | 0.21 [0.03-0.40] | 0.03 | 0.22 [0.03-0.40] | **0.02** |
| Coronary artery disease | 0.21 [-0.01-0.44] | 0.06 | 0.21 [-0.005-0.44] | 0.06 |
| History of myocardial infarction | 0.16 [-0.10-0.42] | 0.22 |  |  |
| Family history of SAH | 0.01 [-0.1-0.1] | 0.85 |  |  |
| Family history of aneurysms | -0.01 [-0.1-0.07] | 0.73 |  |  |
| Aspirin use at diagnosis | -0.02 [-0.17-0.13] | 0.82 |  |  |
| Anticoagulant use at diagnosis | 0.34 [0.01-0.67] | 0.04 |  |  |
| Antihyperlipidemic agent use at diagnosis | 0.08 [-0.05-0.21] | 0.23 |  |  |
|  | | | | |
| **Neck diameter** |  |  |  |  |
| Age at diagnosis | 0.024 [0.015-0.033] | <0.01 | 0.02 [0.02-0.03] | **<0.01** |
| Current alcohol use | -0.080 [-0.33-0.17] | 0.53 |  |  |
| Current tobacco use | -0.050 [-0.31-0.21] | 0.70 |  |  |
| Tobacco use >1 pack per day | 0.34 [-0.12-0.80] | 0.15 |  |  |
| Years since quit tobacco | -0.002 [-0.009-0.004] | 0.46 |  |  |
| Female | -0.17 [-0.42-0.074] | 0.17 |  |  |
| Hypertension | 0.20 [-0.039-0.44] | 0.10 |  |  |
| Atrial fibrillation | -0.004 [-1.15-1.14] | 1.00 |  |  |
| History of ischemic stroke | -0.01 [-0.67-0.64] | 0.97 |  |  |
| Coronary artery disease | 0.15 [-0.63-0.93] | 0.70 |  |  |
| History of myocardial infarction | -0.27 [-1.18-0.63] | 0.55 |  |  |
| Family history of SAH | -0.41 [-0.86-0.040] | 0.07 | -0.42 [-0.86- -0.02] | 0.06 |
| Family history of aneurysms | -0.19 [-0.53-0.14] | 0.26 |  |  |
| Aspirin use at diagnosis | -0.05 [-0.58-0.47] | 0.84 |  |  |
| Anticoagulant use at diagnosis | -0.58 [-1.72-0.56] | 0.32 |  |  |
| Antihyperlipidemic agent use at diagnosis | -0.10 [-0.55-0.36] | 0.68 |  |  |
|  | | | | |
| **Irregularity** |  |  |  |  |
| Age at diagnosis | 0.98 [0.97-1.00] | 0.01 | 0.98 [0.96-0.99] | **<0.01** |
| Current alcohol use | 1.05 [0.72-1.54] | 0.80 |  |  |
| Current tobacco use | 1.40 [0.95-2.07] | 0.09 |  |  |
| Tobacco use >1 pack per day | 1.36 [0.69-2.71] | 0.38 |  |  |
| Years since quit tobacco | 0.99 [0.98-1.01] | 0.33 |  |  |
| Female | 0.87 [0.60-1.27] | 0.48 |  |  |
| Hypertension | 0.78 [0.54-1.14] | 0.20 |  |  |
| Atrial fibrillation | 0.64 [0.11-3.92] | 0.63 |  |  |
| History of ischemic stroke | 1.68 [0.59-4.79] | 0.33 |  |  |
| Coronary artery disease | 1.18 [0.35-3.98] | 0.79 |  |  |
| History of myocardial infarction | 1.65 [0.39-7.08] | 0.50 |  |  |
| Family history of SAH | 0.64 [0.31-1.36] | 0.25 |  |  |
| Family history of aneurysms | 0.83 [0.49-1.41] | 0.49 |  |  |
| Aspirin use at diagnosis | 1.16 [0.51-2.62] | 0.73 |  |  |
| Anticoagulant use at diagnosis | 0.64 [0.11-3.92] | 0.63 |  |  |
| Antihyperlipidemic agent use at diagnosis | 1.03 [0.51-2.09] | 0.93 |  |  |
|  | | | | |
| **Daughter dome** |  |  |  |  |
| Age at diagnosis | 0.98 [0.97-0.99] | <0.01 | 0.98 [0.96-0.99] | **<0.01** |
| Current alcohol use | 1.12 [0.77-1.62] | 0.56 |  |  |
| Current tobacco use | 1.43 [0.98-2.09] | 0.06 |  |  |
| Tobacco use >1 pack per day | 0.83 [0.41-1.67] | 0.60 |  |  |
| Years since quit tobacco | 1.01 [0.99-1.02] | 0.31 |  |  |
| Female | 0.89 [0.62-1.28] | 0.53 |  |  |
| Hypertension | 0.75 [0.52-1.07] | 0.11 |  |  |
| Atrial fibrillation | 2.22 [0.24-20.22] | 0.48 |  |  |
| History of ischemic stroke | 0.68 [0.25-1.91] | 0.47 |  |  |
| Coronary artery disease | 0.44 [0.13-1.48] | 0.18 |  |  |
| History of myocardial infarction | 1.67 [0.33-8.47] | 0.54 |  |  |
| Family history of SAH | 0.83 [0.42-1.64] | 0.59 |  |  |
| Family history of aneurysms | 0.82 [0.49-1.37] | 0.45 |  |  |
| Aspirin use at diagnosis | 1.26 [0.52-3.05] | 0.60 |  |  |
| Anticoagulant use at diagnosis | 0.36 [0.06-2.18] | 0.26 |  |  |
| Antihyperlipidemic agent use at diagnosis | 1.17 [0.55-2.57] | 0.68 |  |  |

**Supplemental Table 2**. Univariate and multivariate regression analyses for transitional morphological properties of anterior communicating artery (ACoA) aneurysms.

|  | **Univariate** | | **Multivariate** | |
| --- | --- | --- | --- | --- |
|  | **Coef [95%CI]** | **P value** | **Coef [95% CI]** | **P value** |
| **Size ratio** |  |  |  |  |
| Age at diagnosis | -0.001 [-0.004-0.002] | 0.63 |  |  |
| Current alcohol use | -0.02 [-0.07-0.1] | 0.71 |  |  |
| Current tobacco use | 0.17 [0.08-0.25] | <0.01 | 0.19 [0.06-0.31] | **<0.01** |
| Tobacco use >1 pack per day | 0.15 [-9.5×10^-4^-0.30] | 0.051 |  |  |
| Years since quit tobacco | -0.002 [-0.005-2.9×10^-5^] | 0.053 |  |  |
| Female | -0.10 [-0.18- -0.02] | 0.02 | -0.18 [-0.30- -0.05] | **<0.01** |
| Hypertension | 0.01 [-0.07-0.09] | 0.83 |  |  |
| Atrial fibrillation | -0.08 [-0.47-0.32] | 0.70 |  |  |
| History of ischemic stroke | 0.11 [-0.12-0.33] | 0.35 |  |  |
| Coronary artery disease | 0.05 [-0.22-0.32] | 0.71 |  |  |
| History of myocardial infarction | 0.06 [-0.25-0.38] | 0.69 |  |  |
| Family history of SAH | -0.10 [-0.25-0.04] | 0.15 |  |  |
| Family history of aneurysms | -0.06 [-0.17-0.05] | 0.25 |  |  |
| Aspirin use at diagnosis | -0.005 [-0.19-0.18] | 0.96 |  |  |
| Anticoagulant use at diagnosis | 0.04 [-0.35-0.44] | 0.83 |  |  |
| Antihyperlipidemic agent use at diagnosis | -0.01 [-0.17-0.14] | 0.87 |  |  |
|  | | | | |
| **Flow Angle** |  |  |  |  |
| Age at diagnosis | -0.25 [-0.40- -0.10] | <0.01 | -0.24 [-0.39- -0.078] | **<0.01** |
| Current alcohol use | -0.73 [-4.7-3.3] | 0.72 |  |  |
| Current tobacco use | 4.1 [-0.028-8.2] | 0.05 |  |  |
| Tobacco use >1 pack per day | 5.31 [-2.05-12.69] | 0.16 |  |  |
| Years since quit tobacco | -0.02 [-0.13-0.10] | 0.78 |  |  |
| Female | -2.9 [-6.9-1.0] | 0.15 |  |  |
| Hypertension | -0.66 [-4.6-3.2] | 0.74 |  |  |
| Atrial fibrillation | -7.95 [-27.29-11.39] | 0.42 |  |  |
| History of ischemic stroke | -1.23 [-12.33-9.88] | 0.83 |  |  |
| Coronary artery disease | 5.65 [-7.57-18.9] | 0.40 |  |  |
| History of myocardial infarction | -8.89 [-24.3-6.48] | 0.26 |  |  |
| Family history of SAH | -0.30 [-7.5-6.9] | 0.94 |  |  |
| Family history of aneurysms | -0.13 [-5.6-5.3] | 0.96 |  |  |
| Aspirin use at diagnosis | -2.13 [-11.0-6.79] | 0.64 |  |  |
| Anticoagulant use at diagnosis | 9.33 [-10.0-28.7] | 0.34 |  |  |
| Antihyperlipidemic agent use at diagnosis | -3.29 [-11.0-4.39] | 0.40 |  |  |

**Supplemental Table 3**. Univariate and multivariate regression analyses for extrinsic morphological properties of anterior communicating artery (ACoA) aneurysms.

|  | **Univariate** | | **Multivariate** | |
| --- | --- | --- | --- | --- |
|  | **Coef [95%CI]** | **P value** | **Coef [95% CI]** | **P value** |
| **Ipsilateral A1** |  |  |  |  |
| Age at diagnosis | 0.003 [0.0001-0.05] | 0.04 |  |  |
| Current alcohol use | -0.078 [-0.15- -0.006] | 0.03 |  |  |
| Current tobacco use | -0.071 [-0.14-0.003] | 0.06 |  |  |
| Tobacco use >1 pack per day | 0.05 [-0.08-0.18] | 0.46 |  |  |
| Years since quit tobacco | 0.002 [-3.2×10^-4^-0.004] | 0.098 | 0.002 [1.9×10^-4^-0.004] | **0.03** |
| Female | -0.013 [-0.084-0.058] | 0.72 |  |  |
| Hypertension | 0.062 [-0.007-0.13] | 0.08 |  |  |
| Atrial fibrillation | 0.08 [-0.27-0.43] | 0.63 |  |  |
| History of ischemic stroke | 0.04 [-0.16-0.24] | 0.72 |  |  |
| Coronary artery disease | 0.26 [0.02-0.49] | 0.03 | 0.27 [0.002-0.53] | **0.048** |
| History of myocardial infarction | 0.07 [-0.20-0.35] | 0.60 |  |  |
| Family history of SAH | -0.062 [-0.19-0.067] | 0.35 |  |  |
| Family history of aneurysms | -0.036 [-0.13-0.061] | 0.47 |  |  |
| Aspirin use at diagnosis | 0.01 [-0.15-0.17] | 0.89 |  |  |
| Anticoagulant use at diagnosis | -0.007 [-0.36-0.34] | 0.97 |  |  |
| Antihyperlipidemic agent use at diagnosis | 0.07 [-0.07-0.21] | 0.34 |  |  |
|  | | | | |
| **Ipsilateral A2** |  |  |  |  |
| Age at diagnosis | 0.005 [0.002-0.008] | <0.01 |  |  |
| Current alcohol use | -0.1 [-0.17- -0.024] | 0.01 | -0.10 [-0.16- -0.04] | **<0.01** |
| Current tobacco use | -0.05 [-0.13-0.028] | 0.21 |  |  |
| Tobacco use >1 pack per day | 0.06 [-0.08-0.19] | 0.44 |  |  |
| Years since quit tobacco | 9.2×10^-4^ [-0.001-0.003] | 0.40 |  |  |
| Female | 0.032 [-0.04-0.11] | 0.39 |  |  |
| Hypertension | 0.070 [-0.002-0.14] | 0.06 |  |  |
| Atrial fibrillation | 0.29 [-0.07-0.64] | 0.11 |  |  |
| History of ischemic stroke | -0.05 [-0.25-0.16] | 0.65 |  |  |
| Coronary artery disease | 0.28 [-0.03=0.52] | 0.03 | 0.29 [0.04-0.53] | **0.02** |
| History of myocardial infarction | 0.02 [-0.27-0.30] | 0.91 |  |  |
| Family history of SAH | -0.08 [-0.21-0.050] | 0.22 |  |  |
| Family history of aneurysms | -0.02 [-0.12-0.078] | 0.68 |  |  |
| Aspirin use at diagnosis | -0.05 [-0.21-0.12] | 0.59 |  |  |
| Anticoagulant use at diagnosis | -0.21 [-0.57-0.14] | 0.24 |  |  |
| Antihyperlipidemic agent use at diagnosis | -0.06 [-0.21-0.08] | 0.38 |  |  |
|  | | | | |
| **Ipsilateral A1-A2 angle** |  |  |  |  |
| Age at diagnosis | -0.38 [-0.5- -0.2] | <0.01 | -0.21 [-0.41- -0.01] | **0.04** |
| Current alcohol use | 0.81 [-3.4-5.0] | 0.70 |  |  |
| Current tobacco use | 2.3 [-2.0-6.6] | 0.30 |  |  |
| Tobacco use >1 pack per day | -4.5 [-12.3-3.22] | 0.25 |  |  |
| Years since quit tobacco | 0.02 [-0.09-0.13] | 0.77 |  |  |
| Female | 4.4 [0.26-8.5] | 0.04 | 5.8 [0.32-11.3] | **0.04** |
| Hypertension | -1.7 [-5.8-2.4] | 0.42 |  |  |
| Atrial fibrillation | 11.8 [-6.83-30.3] | 0.21 |  |  |
| History of ischemic stroke | 6.51 [-4.14-17.2] | 0.23 |  |  |
| Coronary artery disease | -11.2 [-23.9-1.43] | 0.08 |  |  |
| History of myocardial infarction | -7.65 [-22.5-7.16] | 0.31 |  |  |
| Family history of SAH | 3.0 [-4.6-11] | 0.44 |  |  |
| Family history of aneurysms | 5.1 [-0.62-11] | 0.08 |  |  |
| Aspirin use at diagnosis | -0.96 [-9.55-7.63] | 0.83 |  |  |
| Anticoagulant use at diagnosis | 13.2 [-5.39-31.7] | 0.16 |  |  |
| Antihyperlipidemic agent use at diagnosis | 4.68 [-2.70-12.1] | 0.21 |  |  |
|  | | | | |
| **Ipsilateral A2-ACoA angle** |  |  |  |  |
| Age at diagnosis | 0.24 [0.080-0.41] | <0.01 |  |  |
| Current alcohol use | 0.96 [-3.5-5.4] | 0.67 |  |  |
| Current tobacco use | -3.5 [-8.1-1.0] | 0.13 |  |  |
| Tobacco use >1 pack per day | 5.16 [-3.10-13.4] | 0.22 |  |  |
| Years since quit tobacco | 0.11 [-0.002-0.22] | 0.054 | 0.11 [-0.002-0.22] | 0.054 |
| Female | -4.6 [-8.9- -0.24] | 0.04 |  |  |
| Hypertension | -2.2 [-6.4-2.1] | 0.32 |  |  |
| Atrial fibrillation | -12.2 [-31.0-6.64] | 0.20 |  |  |
| History of ischemic stroke | -5.09 [-15.9-5.71] | 0.35 |  |  |
| Coronary artery disease | -6.04 [-18.9-6.84] | 0.36 |  |  |
| History of myocardial infarction | -2.19 [-17.2-12.8] | 0.77 |  |  |
| Family history of SAH | 2.2 [-5.8-10] | 0.60 |  |  |
| Family history of aneurysms | -0.54 [-6.6-5.5] | 0.86 |  |  |
| Aspirin use at diagnosis | -2.25 [-10.9-6.44] | 0.61 |  |  |
| Anticoagulant use at diagnosis | 1.72 [-17.2-20.6] | 0.86 |  |  |
| Antihyperlipidemic agent use at diagnosis | -0.33 [-7.83-7.17] | 0.93 |  |  |
|  | | | | |
| **Contralateral A1-A2 angle** |  |  |  |  |
| Age at diagnosis | -0.55 [-0.73- -0.38] | <0.01 | -0.34 [-0.57- -0.12] | **<0.01** |
| Current alcohol use | -1.9 [-6.7-3.0] | 0.45 |  |  |
| Current tobacco use | 1.0 [-3.9-6.0] | 0.68 |  |  |
| Tobacco use >1 pack per day | -0.73 [-9.58-8.12] | 0.87 |  |  |
| Years since quit tobacco | -0.06 [-0.19-0.06] | 0.32 |  |  |
| Female | 0.61 [-4.2-5.4] | 0.80 |  |  |
| Hypertension | -6.0 [-11- -1.3] | 0.01 |  |  |
| Atrial fibrillation | -17.0 [-35.9-2.02] | 0.08 |  |  |
| History of ischemic stroke | -3.83 [-15.6-7.89] | 0.52 |  |  |
| Coronary artery disease | -3.2 [-18.5-12.0] | 0.68 |  |  |
| History of myocardial infarction | -3.99 [-21.5-13.5] | 0.65 |  |  |
| Family history of SAH | -2.8 [-12- 5.9] | 0.53 |  |  |
| Family history of aneurysms | -2.7 [-9.2-3.7] | 0.41 |  |  |
| Aspirin use at diagnosis | -5.4 [-15.6-4.8] | 0.30 |  |  |
| Anticoagulant use at diagnosis | 2.35 [-22.2-26.9] | 0.85 |  |  |
| Antihyperlipidemic agent use at diagnosis | 2.01 [-6.5-10.5] | 0.64 |  |  |

**Supplemental Table 4.** Reduced anterior communicating artery (ACoA) score for prediction of rupture with the presence of daughter dome excluded.

| **Points** | **Characteristics** |
| --- | --- |
| -1 | Neck diameter > 3.6mm |
| -1 | Ipsilateral A2-ACoA angle > 128 |
| 1 | Flow angle >130 |
| 4 | Aspect ratio > 1.3 |
| -2 to 5 | **Range of possible points** |

**Supplemental Figure 1.** Percent ruptured aneurysms stratified by the reduced predictive anterior communicating artery aneurysm score defined in Supplemental Table 4.
